# Supplementary figures and images for: Orthohantavirus infections in humans and rodents in the Yichun region, China, from 2016 to 2021
Source: PLoS Negl Trop Dis. 2023 Aug 8;17(8):e0011540. doi: 10.1371/journal.pntd.0011540 (PMC10437993; doi:10.1371/journal.pntd.0011540)

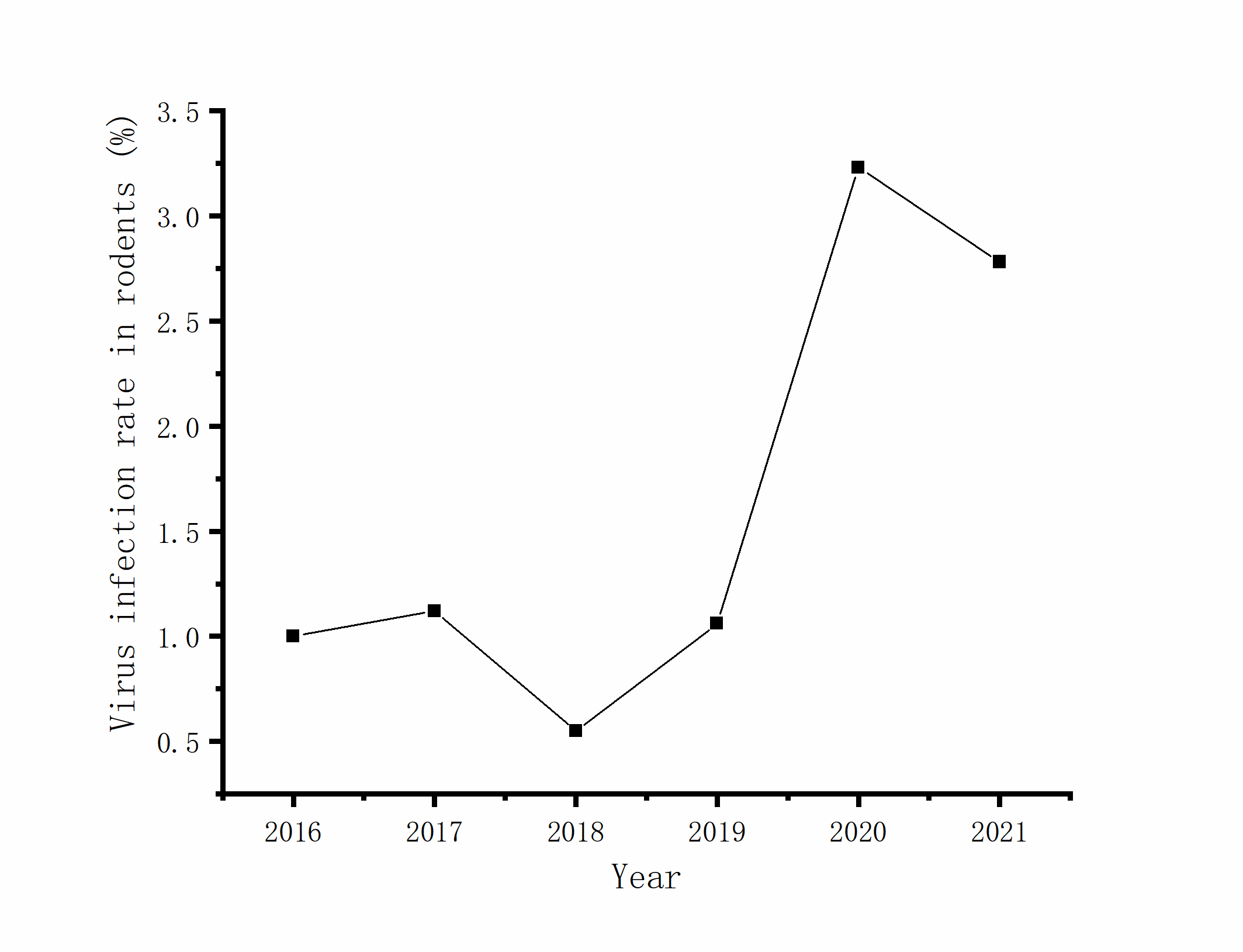

Supplement: S1 Fig — (TIF) [file pntd.0011540.s006.tif]

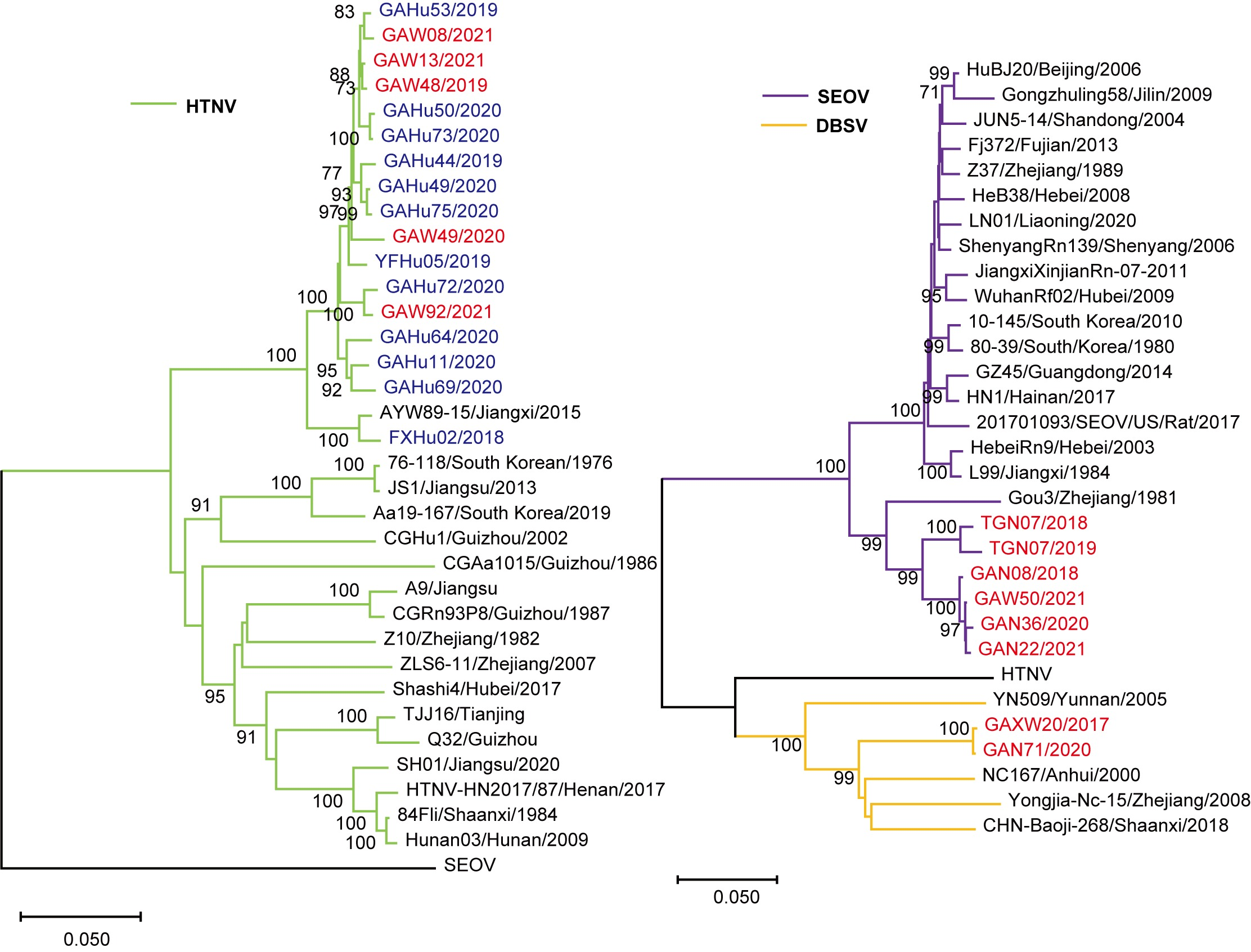

Supplement: S2 Fig — The blue font indicates the virus strain recovered from human specimens in the Yichun region; the red font indicates the virus strain recovered from rodent specimens in the Yichun region. The black font indicates the virus strain from GenBank. Numbers at nodes indicate bootstrap values and only >70% are shown. The scale bars indicate 0.05 substitutions per sit. (TIF) [file pntd.0011540.s007.tif]
