# Supplementary material for: Orthohantavirus infections in humans and rodents in the Yichun region, China, from 2016 to 2021
Source: PLoS Negl Trop Dis. 2023 Aug 8;17(8):e0011540. doi: 10.1371/journal.pntd.0011540 (PMC10437993; doi:10.1371/journal.pntd.0011540)
Supplement: Attachment — Submitted filename: PNTD-D-23-00074_revised1.pdf [file pntd.0011540.s008.pdf]

PLOS Neglected Tropical Diseases

Orthohantavirus infections in humans and rodents in the Yichun region, China, from 2016 to 2021

--Manuscript Draft--

**Manuscript Number:**	PNTD-D-23-00074
**Full Title:**	Orthohantavirus infections in humans and rodents in the Yichun region, China, from 2016 to 2021
**Short Title:**	Orthohantavirus infections in humans and rodents
**Article Type:**	Research Article
**Keywords:**	Orthohantavirus; Hemorrhagic fever with renal syndrome; Hantaan orthohantavirus; Seoul orthohantavirus; Dabieshan orthohantavirus; epidemiology
**Abstract:**	Background<br>Rodents are the predominant natural hosts of orthohantavirus and the source of human infection, hemorrhagic fever with renal syndrome (HFRS) caused by orthohantavirus is a severe public health problem in the Yichun region, Jiangxi Province, China. However, little information is known about the infection of orthohantavirus in humans and rodents, and genetic characteristics of the epidemic orthohantavirus in the region.<br>Methods<br>The clinical data of HFRS cases in 2016–2021 was analyzed. Virus infection in rodents was analyzed by orthohantavirus antigen detection using immunofluorescent assay, and the species of orthohantaviruses in rodents and patients were identified by real-time RT-PCR and gene sequencing. The complete S and partial M segments of orthohantaviruses from rodents and patients were recovered and analyzed.<br>Results<br>A total of 1573 HFRS cases were reported in the Yichun region from 2016 to 2021, including 11 death cases. HFRS cases peaked twice in each year: in winter from November to January and in early summer from May to June. Middle-aged male farmers constituted the predominant population suffering from HFRS. The orthohantavirus antigen was identified in five species of rodents: Apodemus agrarius, Rattus norvegicus, Crocidura attenuate, Rattus losea, and Niviventer confucianus. Real-time RT-PCR test and genetic analysis results showed that Hantaan orthohantavirus (HTNV), Seoul orthohantavirus (SEOV), and Dabieshan orthohantavirus (DBSV) were all circulated in the rodents. HTNV, SEOV, and DBSV from the rodents were distantly related to other known orthohantaviruses and belonged to novel genetic lineages. SEOV and HTNV were found in patients of HFRS, but 97.8% (90/92) of the infections were caused by HTNV. Summer and winter peaks were related to HTNV. The sequences recovered from the HFRS cases belonged to HTNV and closely related to those from Apodemus agrarius.<br>Conclusions<br>In Yichun region, the orthohantaviruses transmitted in rodents includes HTNV, SEOV and DBSV, which have obvious genetic characteristics and high genetic diversity. At the same time, this region is a HFRS mixed epidemic area dominated by HTNV, with two peaks every year, which deserves our high attention.
**Additional Information:**	
**Question**	**Response**
**Government Employee**<br><br>Are you or any of the contributing authors an employee of the United States government?<br><br>Manuscripts authored by one or more US	No - No authors are employees of the U.S. government.

Powered by Editorial Manager® and ProduXion Manager® from Aries Systems Corporation

Government employees are not copyrighted, but are licensed under a CC0 Public Domain Dedication, which allows unlimited distribution and reuse of the article for any lawful purpose. This is a legal requirement for US Government employees.<br><br>This will be typeset if the manuscript is accepted for publication.	
**Financial Disclosure**<br><br>Enter a financial disclosure statement that describes the sources of funding for the work included in this submission and the role the funder(s) played. This includes grants and any commercial funding of the work or authors.<br><br>This statement will be typeset if the manuscript is accepted for publication.<br><br>Review the submission guidelines and the instructions link below for detailed requirements and guidance.	This work was supported by the Key Research and Development Program of Jiangxi Province (20203BBG73055), Science and Technology Program of Jiangxi Health Commission (20221313), the Key Laboratory of Important and Emerging Viral Infectious Diseases of Jiangxi Health Commission. The funders had no role in the study design, data collection and analysis, decision to publish, or the preparation of the manuscript.
**Competing Interests**<br><br>On behalf of all authors, disclose any competing interests that could be perceived to bias this work.<br><br>This statement will be typeset if the manuscript is accepted for publication.<br><br>Review the instructions link below and PLOS NTDs' competing interests policy to determine what information must be disclosed at submission.	The authors have no conflicts of interest to declare.
**Data Availability**<br><br>Provide a **Data Availability Statement** in the box below. This statement should detail where the data used in this	All relevant data are within the manuscript and its Supporting Information files.

submission can be accessed. This statement will be typeset if the manuscript is accepted for publication.<br><br>Before publication, authors are required to make all data underlying their findings fully available, without restriction. Review our PLOS Data Policy page for detailed information on this policy. Instructions for writing your Data Availability statement can be accessed via the Instructions link below.	

Powered by Editorial Manager® and ProduXion Manager® from Aries Systems Corporation

Manuscript Click here to access/download;Manuscript;Manuscript.docx

Orthohantavirus infections in humans and rodents in the Yichun region, China, from 2016 to 2021

Shi-Wen Liu[1, 2], Jian-Xiong Li[2], Long Zou[1], Xiao-Qing Liu[2], Gang Xu[2], Ying Xiong[2]*, Zhong-Er Long[1]*

[1] College of Life Sciences, Nanchang Key Laboratory of Microbial Resources Exploitation & Utilization from Poyang Lake Wetland, Jiangxi Normal University, Nanchang, Jiangxi, China

[2] Laboratory of Viral Infectious Disease, Jiangxi Provincial Center for Disease Control and Prevention, Nanchang, Jiangxi, China

* Corresponding author

E-mail：*xiongying8300087@163.com (Y X); longzhonger@jxnu.edu.cn（ZE L）

Abstract

Background

Rodents are the predominant natural hosts of orthohantavirus and the source of human infection, hemorrhagic fever with renal syndrome (HFRS) caused by orthohantavirus is a severe public health problem in the Yichun region, Jiangxi Province, China. However, little information is known about the infection of orthohantavirus in humans and rodents, and genetic characteristics of the epidemic orthohantavirus in the region.

Methods

The clinical data of HFRS cases in 2016–2021 was analyzed. Virus infection in rodents was analyzed by orthohantavirus antigen detection using immunofluorescent assay, and the species of orthohantaviruses in rodents and patients were identified by real-time RT-PCR and gene sequencing. The complete S and partial M segments of orthohantaviruses from rodents and patients were recovered and analyzed.

Results

A total of 1573 HFRS cases were reported in the Yichun region from 2016 to 2021, including 11 death cases. HFRS cases peaked twice in each year: in winter from November to January and in early summer from May to June. Middle-aged male farmers constituted the predominant population suffering from HFRS. The orthohantavirus antigen was identified in five species of rodents: *Apodemus agrarius*, *Rattus norvegicus*, *Crocidura attenuate*, *Rattus losea*, and *Niviventer confucianu*s. Real-time RT-PCR test and genetic analysis results showed that Hantaan orthohantavirus (HTNV), Seoul orthohantavirus (SEOV), and Dabieshan

orthohantavirus (DBSV) were all circulated in the rodents. HTNV, SEOV, and DBSV from the rodents were distantly related to other known orthohantaviruses and belonged to novel genetic lineages. SEOV and HTNV were found in patients of HFRS, but 97.8% (90/92) of the infections were caused by HTNV. Summer and winter peaks were related to HTNV. The sequences recovered from the HFRS cases belonged to HTNV and closely related to those from *Apodemus agrarius*.

Conclusions

In Yichun region, the orthohantaviruses transmitted in rodents includes HTNV, SEOV and DBSV, which have obvious genetic characteristics and high genetic diversity. At the same time, this region is a HFRS mixed epidemic area dominated by HTNV, with two peaks every year, which deserves our high attention.

Author Summary

Hemorrhagic fever with renal syndrome (HFRS) caused by orthohantavirus is a serious public health problem in the Yichun region in Jiangxi Province, China. However, little information is known about the infection of orthohantavirus in humans and rodents, and genetic characteristics of the epidemic orthohantavirus in the region. To reveal the above problems, we analyzed the epidemiology of HFRS and performed a molecular investigation of orthohantavirus in the Yichun region. During 2016－2021, a total of 1573 HFRS cases were recorded with an average fatality rate of 0.70%. HFRS consistently occurred and peaked in summer and winter in the Yichun region, it was a mixed epidemic area of HFRS dominated by Hantaan orthohantavirus (HTNV). We identified new lineages of Seoul orthohantavirus (SEOV), HTNV and

Dabieshan orthohantavirus (DBSV) from rodent lung samples in the Yichun region, which enlarged the genetic diversity of the orthohantaviruses. The sequences recovered from the HFRS patients belonged to HTNV and closely related to those from rodents, suggesting a direct viral transmission from rodents to humans. The HFRS epidemic in the Yichun region should be closely monitored due to its distinct genetic characteristic and high genetic diversity.

Background

Members of the family Hantaviridae, genus Orthohantavirus, orthohantaviruses are enveloped, single-stranded, and negative sense RNA viruses [1]. The orthohantavirus RNA genome consists of three segments: L (large), M (medium), and S (small), which encode RNA-dependent RNA polymerase (L), glycoproteins (Gn and Gc), and nucleocapsid protein (N), respectively [2]. Orthohantaviruses are carried and transmitted by small animals, including rodents, shews, and bats [3]. Rodents are the predominant natural hosts and the source of human infection [4]. The viruses cause hemorrhagic fever with renal syndrome (HFRS) and cardiopulmonary syndrome, thus raising worldwide concern [5, 6].

HFRS is characterized by fever, acute kidney damage, and hemorrhage manifestations, and the case fatality rates are up to 15% [3, 7]. The majority of the HFRS cases in the world occurred in China. In fact, a total of 1,557,622 HFRS cases were recorded in China from 1950 to 2007. The cases peaked in 1986, when the largest annual number (115,804 cases) of HFRS cases was reported [8]. China has made a comprehensive effort to control and prevent HFRS, successfully decreasing

the incidence rate from 1990 to 2010 [8, 9]. However, it remains a considerable public health threat in China, having 100,000 cases and causing 1116 deaths over the past 10 years [10]. Moreover, the incidence rate has increased drastically in recent years in some parts of China [11]. Several orthohantavirus species can cause HFRS, including Hantaan orthohantavirus (HTNV), Seoul orthohantavirus (SEOV), Puumala orthohantavirus (PUUV), and Dobrava-Belgrade orthohantavirus (DOBV) [12]. HFRS in China is primarily caused by HTNV and SEOV [13].

The Yichun region (located at 27° 33′–29° 06′ N, 113° 54[illegible]16° 27′ E) is one prefectural-level city of Jiangxi Province, China. (Fig 1). A total of 7203 HFRS cases were reported in Jiangxi from 2005 to 2018, and case fatality rate was 1.34% [14]. More than 50% of these cases occurred in the Yichun region [15]. Despite this situation, no genetic investigation of orthohantaviruses has been carried out in the Yichun region. The circulating pathogens of HFRS in humans remain unknown.

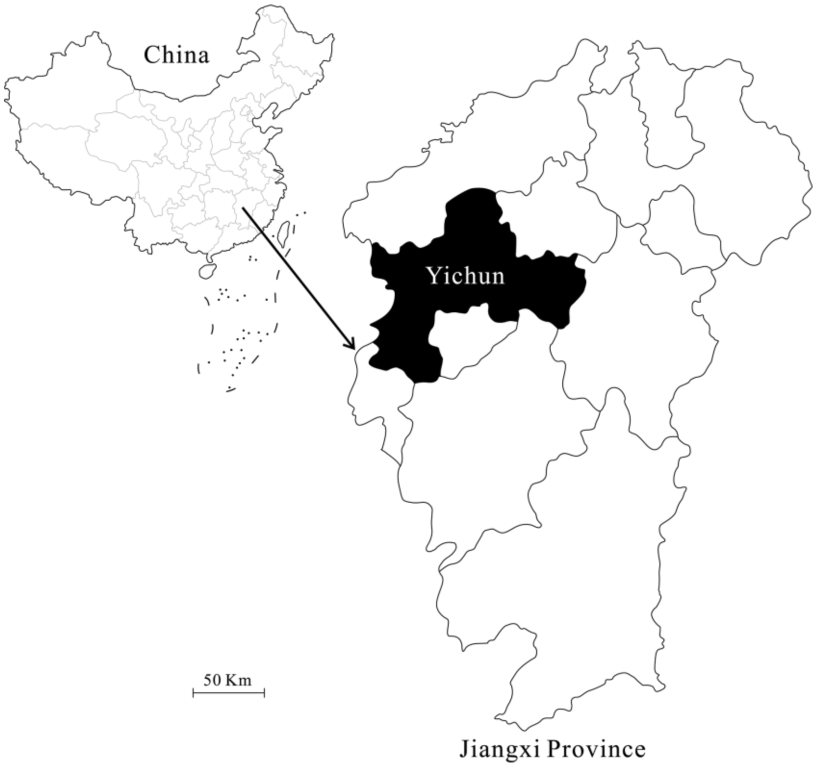

Fig 1. Map of the Yichun region and Jiangxi Province, China.

To understand orthohantavirus infection in humans and rodents in the Yichun region, we analyzed clinical data for HFRS cases from 2016 to 2021. Virus infection in rodents was analyzed by orthohantavirus antigen detection. The orthohantavirus species in rodents and patients were identified by real-time RT-PCR and gene sequencing. The complete S and partial M segments of the orthohantaviruses from rodents and patients were recovered and analyzed. The results will provide important information for HFRS control in the Yichun region and other cities in China.

Materials and Methods

Ethics statement

This study was approved by the ethics committee of Jiangxi Provincial Center for Disease Control and Prevention to ensure the patient's informed consent, confidentiality, and anonymity. Rodents were strictly treated according to the experimental animal welfare and ethical treatment guidelines issued by the Ministry of Science and Technology of China (http://www.most.gov.cn/fggw/zfwj/zfwj2006/200609/t20060930_54389.htm).

HFRS data

The information of the HFRS cases included age, sex, occupation, diagnostic types (laboratory confirmed or clinical diagnostic cases), and incidence rates from 2016 to 2021 were obtained from the China Information System for Disease Control and Prevention (CISDCP). HFRS cases from the CISDCP were reported according to the

Diagnostic Criteria for Epidemic Hemorrhagic Fever (WS278-2008) issued by the National Health Commission of the People's Republic of China (http://www.nhfpc.gov.cn/zwgkzt/s9491/200802/39043.shtml).

Samples

The prevalence, species, and genetic characteristic of orthohantaviruses in rodents were explored. The rodents were trapped in spring and fall/winter seasons by the night trapping method in HFRS endemic areas in the Yichun region from 2016 to 2021. The trap sites included field and residential areas. The trapped rodents were initially classified into specific species by morphological examination and dissected as soon as they were authenticated, and lung tissues were collected.

The species and genetic characteristics of orthohantaviruses in humans were also explored. Serum samples were collected from patients with HFRS in the epidemic area in the Yichun region, which included Gaoan, Yifeng, Shanggao, and Fengxin counties. The patients were diagnosed by the presence of special IgM antibodies against orthohantaviruses. Serum samples from 92 patients were collected from 2017 to 2020.

Detection of orthohantaviral antigen in rodents

Orthohantaviral antigens in rodent lung samples were detected by immunofluorescent assay as described previously [16]. Lung samples were sliced and mounted on slides by a cryomicrotome, dried in a bio-safety cabinet, fixed with cold acetone for 20 min, and air-dried. FITC-labeled anti-SEOV/HTNV antibodies (10 μL; provided by the Fourth Military Medical University) was added to each sample. The slides were

incubated in a 37 °C water bath for 45 min, washed with 1× PBS buffer three times and then distilled water once, blow-dried, and sealed. The slides were observed under a fluorescence microscope at 100× magnification. The mitochondrial *cytochrome b* gene of rodents with the orthohantavirus antigen were recovered using the PCR primers MVZ04 and MVZ05 according to a previously described method [17]. The species of the rodents were confirmed by *cytochrome b* sequence analysis.

Extraction of total RNA

The rodents' lung samples with the orthohantavirus antigen were confirmed by immunofluorescent assay and mechanically homogenized by a bullet blender (Next advance in the United States), and then centrifuged at 8000 *g* for 10 min. The supernatant of each tissue suspension was collected and used for nucleic acid extraction. Total RNA from supernatants and 92 serum samples from humans were extracted with QIAamp viral RNA mini kit (QIAGEN). Nucleic acid was dissolved with 40 μL of nuclease-free water and stored at −80 °C.

Real-time RT-PCR

Real-time RT-PCR for HTNV and SEOV was performed as previously described [18]. Total RNA from rodents and patients were reversed and quantitatively analyzed using an AgPath-ID one-step RT-PCR kit (Thermo Fisher Scientific, USA). The PCR primers sequences and probe sequences:

HTNV-forward: 5′-GACAACAAAGGGACTAGA-3′,

HTNV-reverse: 5′-GTGCATTGGGTAGAGATA-3′,

HTNV-Probe: 5′-FAM-ATTCGGTTCAAGGATGATAGC-BHQ1-3′,

SEOV-forward: 5′-TGGCTTCAAAAACTGTGG-3′,

SEOV-reverse: 5′-GTCCAGTTGTATTCCCAT-3′,

SEOV-Probe: 5′VIC-TGATTGTGTGCGTCTGAGGT-BHQ2-3′).

All primers sequences and probe sequences were provided by Sangon Biotech Co, Ltd (Shanghai, China). Real-time RT-PCR reactions were performed on an ABI7500 system (Applied Biosystems Instruments, USA). The procedures were as follows: reverse transcription at 45 °C for 10 min, initial denaturation at 95 °C for 10 min, 45 cycles at 95 °C for 15 s, and 55 °C for 45 s. The sample with a Ct value of less than 40 for at least 1 of the replicates was considered positive for orthohantavirus.

Partial M and complete segment sequencing

Partial M genes were amplified by a nested PCR protocol including two amplification rounds. A PrimeScript one-step RT-PCR kit (TAKARA, Dalian) was used in the first round, and procedures were as follows: an initial step for cDNA synthesis at 50 °C for 30 min, denaturation at 94 °C for 2 min, 35 cycles of 94 °C for 30 s, 52 °C for 30 s, 72 °C for 1 min, and final extension at 72 °C for 10 min. Total RNA was used as the template with primers:

HV-F 5′-AAAGTAGGTGITAYATCYTIACAATGTGG-3′,

HV-R 5′-GTACAICCTGTRCCIACCCC-3′.

Amplification was performed in 25 μL volumes each containing 12.5 μL of buffer, 1 μL of enzyme mix, 5 μL of template, 0.5 μL for each primer (20 μmol/L), and 5.5 μL of nuclease-free water. The products of first round were used as templates for the

second round of amplification using 2× GoTaq Green Master Mix (Promega, USA) with the following primers [19]:

HTNV-F 5′-GAATCCATACTGTGGGCTGCAAGTGC-3′,

HTNV-R 5′-GGATTACAACCCCAGCTCGTCT-3′,

SEOV-F 5′-GTGGACTCTTCTTCTCATTATT-3′,

SEOV-R TGGGCAATCTGGGGGGTTGCATG.

PCR reactions were performed in 50 μL volumes, containing 25 μL of GoTaq Green Master Mix, 5 μL of template, 1 μL of each primer (20 μmol/L), and 18 μL of nuclease-free water. The procedures were as follows: denaturation at 95 °C for 2 min; 35 cycles of 95 °C for 30 s, 55 °C for 30 s, and 72 °C for 40 s; and final extension at 72 °C for 5 min.

The complete S segment gene was amplified using PrimeScript one-step RT-PCR kit (Takara, Dalian, China) with the following primers:

HV-SF (TAGTAGTAGACTCCCTAAAGA),

HV-SR (TAGTAGTAGTATGCTCCCTAA).

PCR reactions were performed in 50 μL volumes each containing 25 μL of buffer, 2 μL of enzyme mix, 5 μL of template, 1 μL of each primer (20 μmol/L), and 16 μL of nuclease-free water. PCR was performed at 50 °C for 30 min for cDNA synthesis, denaturation at 94 °C for 2 min; 35 cycles of 94 °C for 30 s, 55 °C for 30 s, and 72 °C for 1 min and 40 s; and final extension at 72 °C for 10 min. The PCR products of the complete S segment were purified and ligated into pESI-T vector and transformed into *Escherichia coli* DH5α competent cells according to the manufacturer’s

instructions (TaKaRa, Dalian, China). The presence of an insert was confirmed by PCR and agarose gel electrophoresis. The recombinant pESI-T vector was extracted by SK8191 UNIQ-10 plasmid extraction kit (Sangon, Shanghai, China) according to the manufacturer's instructions.

The PCR products of the partial M segment and recombinant pESI-T vector were sequenced by Sangon Biotech Co. (Shanghai, China).

Genetic analysis

The genomic sequences were assembled with Seqman in Lasergene software. Sequences were aligned using the ClustalW method in MEGA X. A sequence identity matrix was obtained with BioEdit version 7.2.5. Phylogenetic analysis was performed using the the neighbor-joining method in MEGA X with a bootstrap analysis of 1000 iterations [20].

Results

Epidemiological characteristics of HFRS

In 2016–2021, a total of 1573 HFRS cases were reported in the Yichun region (Fig 2A), including 11 death cases. The incidence, mortality, and fatality rates for HFRS in the Yichun region in this period are shown in Table 1. HFRS peaked twice in each year (Fig 2B). The early summer peak occurred from May to June, and the winter peak occurred from November to January. The peak in winter was higher than that in summer. Of the 11 deaths, three occurred in January, two occurred in November, two occurred in December, one occurred in March, one occurred in May, one occurred in

June, and one occurred in September. The incidence rate ranged from 2.25 to 6.34 per 100,0000 persons annually, and a declining trend was observed after 2018 (Fig 2C). The average fatality rate was 0.70%.

Table 1. Epidemics of HFRS in the Yichun region (201[illegible]21)

Year	No. of cases	No. of deaths	Incidence rate (per 100,000)	Mortality (per 100,000)	Fatality (%)
2016	311	1	5.64	0.018	0.32
2017	278	1	5.02	0.018	0.36
2018	352	5	6.34	0.09	1.42
2019	288	1	5.17	0.018	0.35
2020	218	3	3.91	0.054	1.38
2021	126	0	2.25	0	0

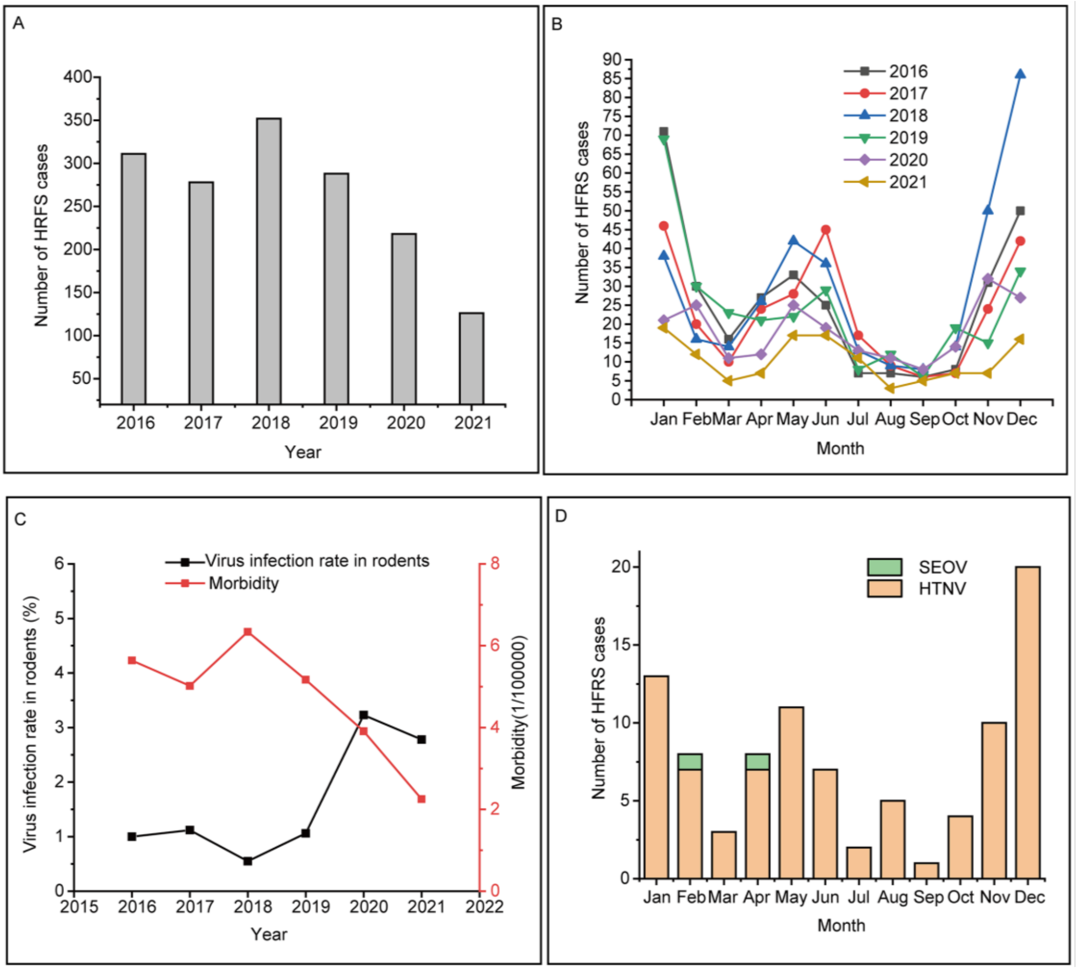

Fig 2. Orthohantavirus infections in rodents and humans in the Yichun region (2016–[illegible]21).

A: Number of HFRS cases in the Yichun region from 2016 to 2021. B: Monthly distribution of HFRS

cases reported on CISDCP. C: The virus infection rate in rodents (black) and HFRS mortality in humans (red). D: Monthly distribution of orthohantavirus from 92 patients with HFRS.

Demographic distribution of HFRS cases

The demographic distribution of HFRS cases in the Yichun region is shown in Table 2. Approximately 63.4% (998/1573) of the cases were males, and thus the male-to-female ratio was 1.74:1. The cases were divided into three age groups: 0–15 years, 16–60 years, and older than 60 years. We found that 59.7% (939/1573) of the cases were 16–60 years. Regarding occupations, farmers accounted for 65.3% (1027/1573), students accounted for 12.5%, and unemployed population accounted for 8.5%.

Table 2. Demographic distribution of HFRS cases in the Yichun region (2016–2021)

Groups	Sex		Age (years)			Occupation			
	Male	Female	0–15	16–60	>60	Farmers	Students	Unemployed population	Others
Number of cases	998	575	223	939	411	1027	197	134	215
Proportion in groups (%)	63.4	36.6	14.2	59.7	26.1	65.3	12.5	8.5	13.7

Species of orthohantaviruses in patients

The species of orthohantaviruses in patients were identified by real-time PCR. Approximately 97.8% (90/92) of the selected 92 HFRS cases were caused by HTNV; and 2.2% (2/90), by SEOV. The onset times in the patients were distributed in 12 months in each year. HFRS cases infected with HTNV occurred throughout the year, and the two cases of SEOV occurred in February and April (Fig 2D). Two peaks were observed in May and December, which were caused by HTNV (Fig 2D).

Virus infection in rodents

A total of 1744 rodents were trapped in the Yichun region from 2016 to 2021 (Table 3). The predominant rodent species were *Apodemus agrarius* (40.2%, 701/1744) and *Rattus norvegicus* (34.9%, 609/1744). Viral infection in rodents was analyzed by orthohantavirus antigen detection. The orthohantavirus antigen was detected in the lungs of 1.72% (33/1744) trapped rodents: 15 *Apodemus agrarius*, 9 *Rattus norvegicus*, 2 *Rattus losea*, 3 *Crocidura attenuate*, and 4 *Niviventer confucianus*. *Mus musculus* and *Rattus flavipectus* were tested negative to orthohantaviruses. The virus infection rate in the rodents showed a rising trend after 2018 (Fig 2C) and reached 3.5% (8/228) in 2021.

Table 3. Prevalence of orthohantavirus infection in trapped rodents in the Yichun region city (2016–2021)*.

Species	Year						Total
	2016	2017	2018	2019	2020	2021	
Apodemus agrarius	84/1	107/4	130/0	165/1	148/4	67/5	701/15
Rattus norvegicus	90/1	114/1	149/2	132/1	44/2	80/2	609/9
Rattus losea	17/0	33/1	40/0	29/0	17/0	59/1	195/2
Mus musculus	10/0	5/0	15/0	29/0	7/0	52/0	118/0
Crocidura attenuate	0/0	7/1	21/0	14/2	8/0	15/0	65/3
Rattus flavipectus	0/0	0/0	0/0	4/0	19/0	5/0	28/0
Niviventer confucianus	0/0	2/2	8/0	3/0	5/2	10/0	28/4
Total	201/2	268/9	363/2	376/4	248/8	228/8	1744/33

*Data showing the number of rodents trapped/number of antigen pos[illegible]e

No significant differe[illegible] in virus infection rate was found between *Apodemus agrarius* and *Rattus norvegicus*. The virus infection rates were 1.37 % (10/728) in residential rodents and 2.26% (23/1016) in field rodents (Table 4), showing no

significant difference ($\chi 2$ = 1.810, P > 0.05). The virus infection rate in rodents in spring and fall/winter were 1.99 % (18/905) and 1.79 % (15/839), respectively (Table 4), showing no significant difference ($\chi 2$ = 0.95, P > 0.05). However, the infection rates for *Rattus norvegicus* in spring (8/310, 2.58 %) and fall/winter (1/299, 0.33 %) differed significantly ($\chi 2$ = 5.274, P = 0.038).

Table 4. Prevalence of orthohantvirus in rodents by trapped area, season, and virus species*

Species	Area		Time	
	Field	Residential area	Spring	Fall/Winter
Apodemus agrarius	701/15/12 (HTNV)	0/0/0	343/8/7 (HTNV)	358/7/5 (HTNV)
Rattus norvegicus	49/1/1 (SEOV)	560/8/7 (SEOV)	310/8/8 (SEOV)	299/1/0
Rattus losea	177/2/1 (SEOV)	18/0/0	107/1/1 (SEOV)	88/1/0
Mus musculus	118/0/0	0/0/0	64/0/0	54/0/0
Crocidura attenuate	53/2/0	10/1/0	37/0/0	26/3/0
Rattus flavipectus	26/0/0	2/0/0	25/0/0	3/0/0
Niviventer confucianus	17/3/3 (2 DBSV+1 SEOV)	11/1/1 (DBSV)	18/1/1 (SEOV)	10/3/3 (DBSV)
Total	1016/23/17	728/10/8	905/18/17	839/15/8

* Data show number of rodents trapped/number of antigen positive/number of PCR positive (virus species)

~~Species of orthohantavirus in rodents~~

In 33 orthohantavirus antigen-positive samples, the virus species were identified in 25 rodents samples by real-time RT-PCR or gene sequencing (Table 4). There were eight samples failed in the virus species identification because of lack of samples or improper storage before reception in our laboratory. HTNV and Dabieshan orthohantavirus (DBSV) were identified in *Apodemus agrarius and Niviventer*

confucianus, respectively. SEOV was identified in *Rattus norvegicus*, *Rattus losea*, and *Niviventer confucianus*.

Genetic characteristics of orthohantaviruses in rodents and humans

To understand the genetic characteristics of orthohantaviruses circulating in rodents and humans, we recovered the complete S and partial M segments (nt 1970–2329) from rodents lung samples and patients' serum samples. Twenty-nine complete S segments from 17 rodents and 12 patients were recovered. Sixty-six partial M segments (2002–2301) from 18 rodents and 48 patients were also recovered. Sequence information was listed in S3 Table. The *cytochrome b* gene sequences of rodents were deposited into GenBank under accession no. OQ187773-OQ187792.

Genetic analysis showed that 19 of the complete S segments of HTNV recovered from 7 *Apodemus agrarius* and 12 patients shared 95.0%–100% nucleotide identities. They shared the highest number of nucleotide identities with HTNV strain AYW89-15 in Jiangxi (94.3%–98.6%) and with HTNV strains from another area (82.3%–85.6%). Seven complete S segments of SEOV recovered from six *Rattus norvegicus* and one *Rattus losea* in the Yichun region shared 95.0%–100% nucleotide identities and 87.2%–90.6% with other known SEOV strains. Three complete S segments of DBSV recovered from *Niviventer confucianus* shared 99.7%–100% nucleotide identities and 83.8%–89.6% with other known DBSV strains.

Fifty-eight partial M segments of HTNV recovered from 10 *Apodemus agrarius* and 48 patients shared 92.3%–100% nucleotide identities with one another, 92.9%–

99.3% with strain AYW89-15, and 79.2%–88.6% with HTNV strains from other areas. Five partial M segments of SEOV in Yichun region showed 92.6%–100% with one another and 83.6%–88% with other known SEOV strains. Three partial M segments of DBSV shared 98.9%–100% nucleotide identities with one another and 85.6%–89.9% with other known DBSV strains. The complete S or partial M segments of SEOV and DBSV showed the highest nucleotide identities (with 9.4%–12.0% divergence) with the strains Gou3 and Wencheng-Nc-469 in Zhejiang Province, respectively.

Phylogenetic trees were constructed with the S and partial M segments of orthohantaviruses. Identical sequences in this study were excluded. In the S segment tree (Fig 3), 25 sequences in the Yinchun region were shown, including 17 of HTNV, 6 of SEOV and 2 of DBSV. Sixteen sequences of the HTNV strains recovered from humans and rodents were distantly related to those of other HTNV strains and formed a distinct lineage. Another HTNV strain (FXHu02/2018) belonged to the lineage of AYW89-15 strain isolated in another part of Jiangxi province [21]. Six SEOV sequences in the Yichun region clustered into two separate groups and distantly related to other SEOV in the S segment tree (Fig 3). Two DBSV sequences in this study belonged to a new lineage (Fig 3).The DBSV strain in the Yichun region were distantly related to those from Shaanxi, Zhejiang, and Anhui Provinces and Yunnan Province, China (Fig 3).

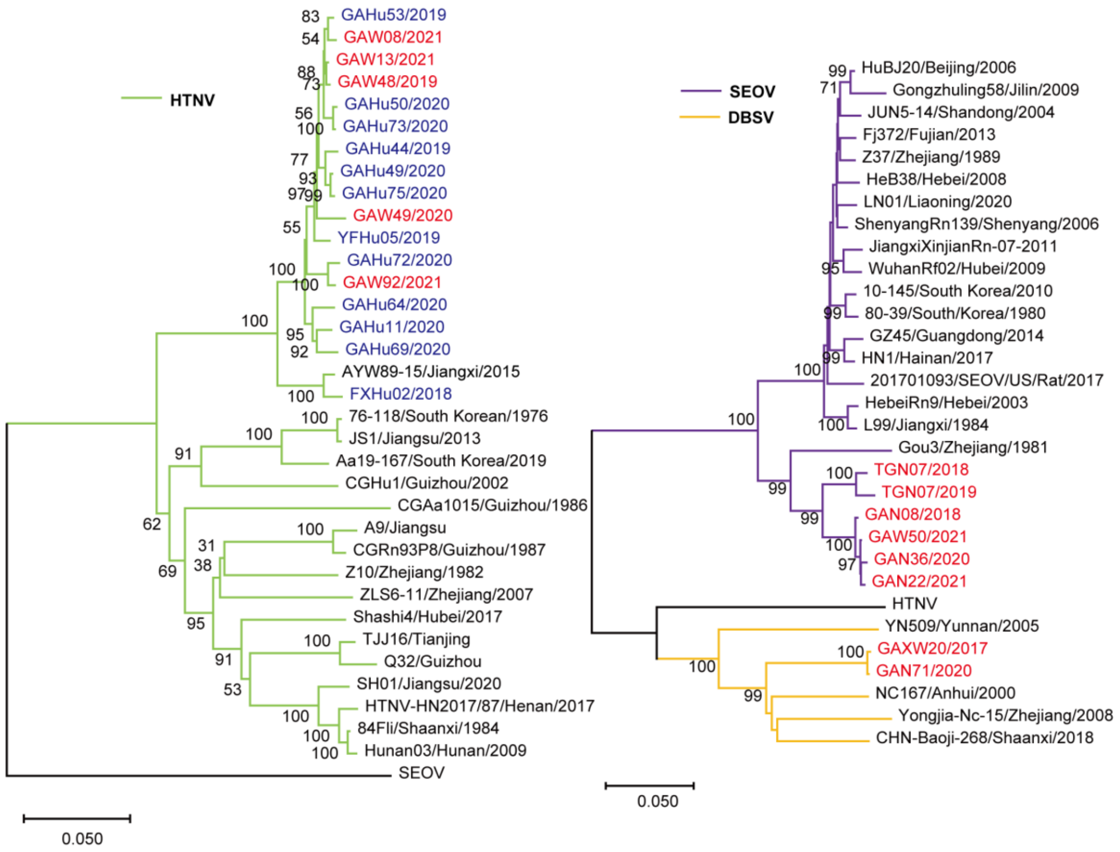

Figure 3. Phylogenetic tree of orthohantavirus based on the CDS region of S segment. The blue font indicates the virus strains recovered from human specimens in the Yichun region; the red font indicates the virus strains recovered from rodent specimens in the Yichun region. The black font indicates the virus strains from GenBank. Numbers at nodes indicate bootstrap values, and only >70% are shown. The scale bars indicate 0.05 substitutions per sit.

Fig 4 shows 17 sequences from humans and 10 sequences from rodents in the Yichun reagion in the M segment tree, including 21 of HTNV, 4 of SEOV and 2 of DBSV. Analysis of the HTNV partial M gene revealed that 21 sequences in this study clustered into at least three separate groups by HTNV genotype: SGHu20/2020 from Shanggao county formed a distinct lineage. FXHu02/2018 from Fengxin county was closely related to the AYW89-15 strain, and other 19 sequences from Gaoan and

Yifeng county formed a novel lineage. In the phylogenetic tree of M segment, the SEOV sequences in the Yichun region also clustered into two separate groups (Fig 4). The DBSV sequences in this study in Fig 4 belonged to a new lineage as in the Fig 3.

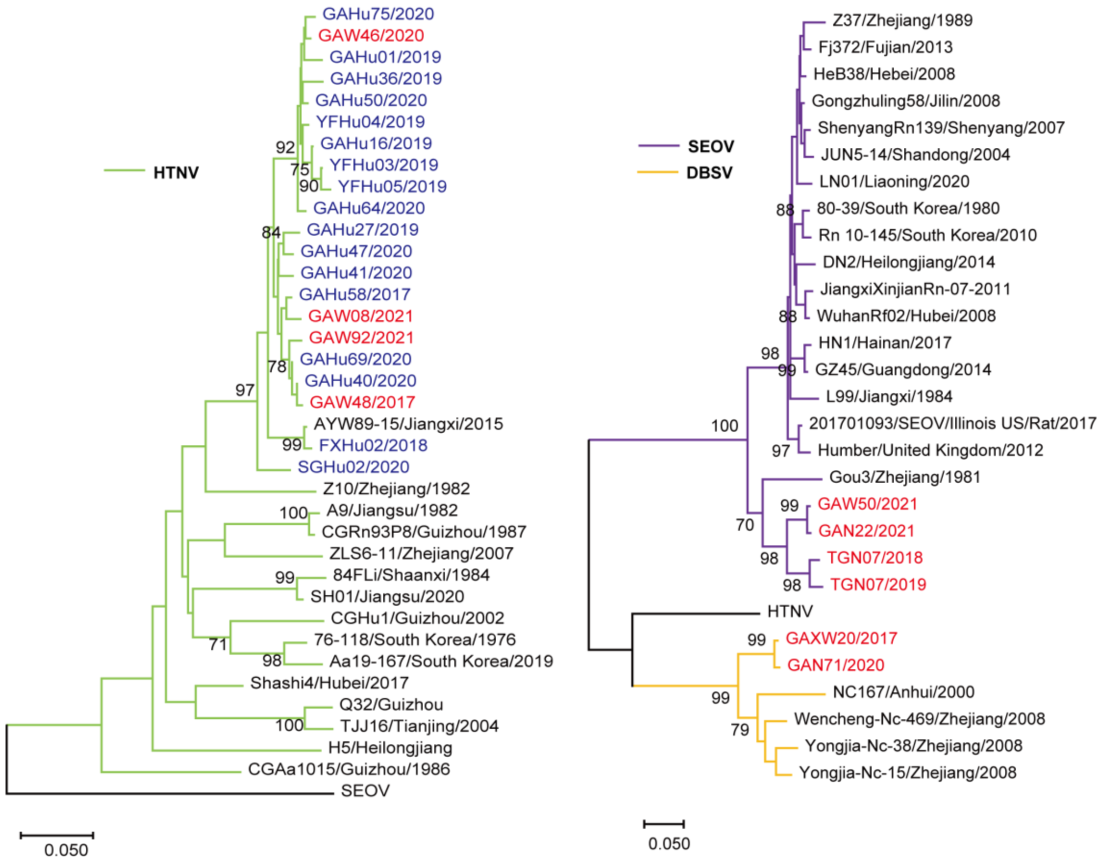

Fig 4. Phylogenetic tree of orthohantavirus based on partial M segment (2002 nt–2301 nt). The blue font indicates the virus strains recovered from human specimens in the Yichun region; the red font indicates the virus strains recovered from rodent specimens in the Yichun region. The black font indicates the virus strains from GenBank. Numbers at nodes indicate bootstrap values, and only >70 % are shown. The scale bars indicate 0.05 substitutions per site.

Discussion

From 2016 to 2021, the morbidity of HFRS in the Yichun region showed a trend of increasing initially and then decreasing, exhibiting a peak in 2018. The lower

epidemic of HFRS in 2020 and 2021 might have been due to the strict control measures for COVID-19 [22]. Given that the COVID-19 epidemic control measures become increasingly optimized to return normalcy to life and the virus infection rate of rodents showed a rising trend after 2018, more HFRS cases may occur in the next few years, so attention should be paid to the epidemic of HFRS in the Yichun region.

HFRS cases occurred monthly with a minor peak in early summer and a major peak in winter in the Yichun region. Male patients with HFRS were higher in number in all age groups, patients 16–60 years old and farmers were the predominant populations. Middle-aged male farmers constituted the predominant population in the Yichun region. Hence, effective vaccination and hygiene in these populations should be strengthened, and protective measures in agricultural operations should be implemented to reduce the incidence rate of HFRS. Epidemiologic investigations had found that peaks in spring and fall/winter were caused by HTNV and SEOV, respectively [23]. However, HTNV is the sole source of HFRS in Xi'an and Baoji, China, winter and summer peaks of HFRS cases have been reported [16, 24]. In the present study, virus species test in patients showed that the winter and summer peaks were all related to HTNV, indicating that the seasonal characteristics of HFRS not only was caused by virus species but also may have been caused by other factors, such as difference in intensity of contact between orthohantavirus hosts and humans and climate change [25, 26].

According to the survey results covering the period 2016–2021, *Apodemus agrarius* and *Rattus norvegicus* were the major carriers of orthohantaviruses in

rodents, and the orthohantavirus antigen was detected from three other rodent species (*Rattus losea*, *Crocidura attenuate*, and *Niviventer confucianus*). The real-time RT-PCR test and genetic analysis results showed that HTNV, SEOV, and DBSV cocirculated in rodents in the Yichun region. The results showed that *Apodemus agrarius* and *Niviventer confucianus* were the hosts of HTNV and DBSV, respectively. SEOV was identified in *Rattus norvegicus*, *Rattus losea*, and *Niviventer confucianus*, showing the wide hosts of SEOV. The sequences recovered from HFRS cases were closely related to those from *Apodemus agrarius*, suggesting that HTNV is directly transmitted from rodents to humans. Interestingly, SEOV and HTNV were found in patients, but more than 97% these patients were infected by HTNV, revealing that the Yichun region is a mixed epidemic area of HFRS dominated by HTNV. The two cases of HFRS infected by SEOV in this study occurred in spring (February and April, respectively), thus may relate to the higher infection rates for *Rattus norvegicus* in spring. The infection rates for *Apodemus agrarius* with HTNV and *Rattus norvegicus* with SEOV showed no significant difference, *Rattus norvegicus* is widely distributed in residential areas and may thus increase the probability of humans infection. However, few patients were infected with SEOV. The possible reason was that the symptom of SEOV infection is mild and is thus often neglected [14, 27]. We should strengthen the surveillance of SEOV in patients to determine its epidemic status in humans.

At least nine genetic lineages of HTNV are endemic in East Asia, including China [28]. HTNV strains belonging to novel lineages have been reported in China,

including AWY89-15, which was isolated from other places of Jiangxi province [21, 39, 30]. In the current study, only one strain (FXHu02/2018) clustered together with AWY89-15, and other HTNV strains formed two new lineages by area in the M segment tree (Fig 4). HTNV genes from the same region, whether from rodents or humans, clustered on the same lineage. These results suggested the unique characteristic and genetic diversity of HTNV in the Yichun region.

The SEOV sequences recovered in this study were closely related, the nucleotide sequence difference of the complete S and partial M segments were less than 5% and 7%, respectively. They clustered together and formed two new distinct lineages in the phylogenetic trees. These sequences shared low identity (≤88.0% nucleotide for complete S gene and ≤90.2 nucleotide for partial M gene) with known SEOV strains, including those (L99, JiangxiXinjianRn-07-2011) from other parts of Jiangxi Province. SEOV is a worldwide endemic orthohantavirus [31]. Most known SEOV strains are genetically homogeneous and do not show geographic clustering, and few SEOV strains differed from most SEOV strains by up to 15% in nucleotides [1]. The SEOV sequences recovered in the Yichun region enlarged the genetic diversity of SEOV, and thus SEOV infections in Yichun region should not be neglected.

DBSV, first identified in Anhui province of China, is a novel species in the genus *Orthohantavirus* [19]. It is carried by *Niviventer confucianus*, which are widely distributed in China. The virus has been detected in *Niviventer confucianus* in Yunnan, Zhejiang, and Shaanxi Provinces, China [25, 32, 33]. In the present study, DBSV was detected in *Niviventer confucianus* in 2017 and 2020, indicating that DBSV continued

to be prevalent in the Yichun region. The identification of DBSV in the Yichun region enlarged its geographical distribution. Given that *Niviventer confucianus* is widely distributed in China, DBSV will probably be found in other places of China. The pathogenicity of DBSV is unclear, attentions should also be paid to DBSV for its high similarity with HTNV and wide geographical distribution [25].

A limitation of this study is that we attempted to amplify orthohantaviral genome sequences from all antigen-positive rodents or human serum samples. Some were not successfully amplified probably because of lack of samples, long delay of operation after the date of onset, low virus load, and improper storage before reception in our laboratory.

Conclusion

This work showed that HFRS consistently occurred in the Yichun region and peaked in the summer and winter. Three orthohantaviruses: HTNV, SEOV, and DBSV circulated in the rodents and belonged to novel genetic lineages. The Yichun region was a mixed epidemic area of HFRS dominated by HTNV. The HFRS epidemic in the Yichun region should be closely monitored due to its distinct genetic characteristic and high genetic diversity.

Acknowledgments

We thank all the municipality CDCs of the Yichun region in the investigation of the HFRS epidemic. We also thank Jun Zhou, Yanni Zhang and Yong Shi for the helps of

data collection and experiments.

Author Contributions

Formal analysis: Shi-Wen Liu,Ying Xiong, Zhong-Er Long.

Funding acquisition: Shi-Wen Liu, Ying Xiong, Zhong-Er Long.

Investigation: Shi-Wen Liu, Long Zou, Xiao-Qing Liu, Gang Xu.

Methodology: Shi-Wen Liu, Jian-Xiong Li, Long Zou, Xiao-Qing Liu,Ying Xiong.

Project administration: Jian-Xiong Li,Ying Xiong, Zhong-Er Long.

Resources: Jian-Xiong Li, Ying Xiong.

Software: Jian-Xiong Li, Gang Xu.

Supervision: Jian-Xiong Li, Ying Xiong, Zhong-Er Long.

Validation: Xiao-Qing Liu, Long Zou.

Visualization: Shi-Wen Liu, Jian-Xiong Li, Long Zou.

Writing – original draft: Shi-Wen Liu, Xiao-Qing Liu, Gang Xu.

Writing – review & editing: Shi-Wen Liu, Long Zou, Ying Xiong, Zhong-Er Long.

Reference

1. Zhang YZ, Lin XD, Shi NF, Wang W, Liao XW, Guo WP, et al. Hantaviruses in small mammals and humans in the coastal region of Zhejiang Province, China. J Med Virol. 2010; 82(6): 987-995. https://doi.org/10.1002/jmv.21737 PMID: 33075097

2. Kim WK, Kim JA, Song DH, Lee D, Kim YC, Lee SY, et al. Phylogeographic

analysis of hemorrhagic fever with renal syndrome patients using multiplex PCR-based next generation sequencing. Sci Rep. 2016; 6: 26017. https://doi.org/10.1038/srep26017 PMID: 27221218

3. Avšič-Županc T, Saksida A, Korva M. Hantavirus infections. Clin Microbiol Infect. 2019; 21S: e6-e16. https://doi.org/10.1111/1469-0691.12291 PMID: 24750436

4. Schönrich G, Rang A, Lütteke N, Raftery MJ, Charbonnel N, Ulrich RG. Hantavirus-induced immunity in rodent reservoirs and humans. Immunol Rev. 2008; 225: 163-189. https://doi.org/10.1111/j.1600-065X.2008.00694.x PMID: 18837782

5. Zhang YZ, Zhang FX, Wang JB, Zhao ZW, Li MH, Chen HX,et al. Hantaviruses in rodents and humans, Inner Mongolia Autonomous Region, China. Emerg Infect Dis. 2009; 15(6): 885-891. https://doi.org/10.3201/eid1506.081126 PMC272735

6. Kang C, Kim JI, Lee J, Bae S, Kim MJ, Song KJ, et al. Human infection with Seoul orthohantavirus in Korea, 2019. PLoS Negl Trop Dis. 2021; 15(2): e0009168. https://doi.org/10.1371/journal.pntd.0009168 PMID: 33617577

7. Zhang S, Wang SW, Yin WW, Liang MF, Li JD, Zhang QF, et al. Epidemic characteristics of hemorrhagic fever with renal syndrome in China, 2006-2012. BMC Infect Dis. 2014; 14: 384. https://doi.org/10.1186/1471-2334-14-384 PMID: 25012160

8. Zhang YZ, Zou Y, Fu ZF, Plyusnin A. Hantavirus infections in humans and animals, China. Emerg Infect Dis. 2010; 16(8): 1195-1203. https://doi.org/10.3201/eid1608.090470 PMID: 20678311

9. Zhang R, Mao ZY, Yang J, Liu SL, Liu Y, Qin SW, et al. The changing epidemiology of hemorrhagic fever with renal syndrome in Southeastern China during 1963-2020: A retrospective analysis of surveillance data. PLoS Negl Trop Dis. 2021; 15(8): e0009673. https://doi.org/10.1371/journal.pntd.0009673 PMID: 34358248
10. Khan A, Khan M, Ullah S, Wei DQ. Hantavirus: The next pandemic we are waiting for? Interdiscip Sci. 2021; 13(1): 147-152. https://doi.org/10.1007/s12539-020-00413-4 PMID: 33486690
11. Yu PB, Tian HY, Ma CF, Ma CA, Wei J, Lu XL, et al. Hantavirus infection in rodents and haemorrhagic fever with renal syndrome in Shaanxi province, China, 1984-2012. Epidemiol Infect. 2015; 143(2): 405-411. https://doi.org/10.1017/S0950268814001009 PMID: 24787374
12. Jiang H, Du H, Wang LM, Wang PZ, Bai XF. Hemorrhagic fever with renal syndrome: pathogenesis and clinical picture. Front Cell Infect Microbiol. 2016; 6: 1. https://doi.org/10.3389/fcimb.2016.00001 PMID: 26870699
13. Liu SY, Wei YM, Han X, Cai Y, Han ZY, Zhang YB, et al. Long-term retrospective observation reveals stabilities and variations of hantavirus infection in Hebei, China. BMC Infect Dis. 2019; 19(1): 765. https://doi.org/10.1186/s12879-019-4402-8 PMID: 31477045
14. Yang S, Gao Y, Liu XB, Liu XQ, Liu YQ, Metelmann S, et al. Spatiotemporal dynamics of hemorrhagic fever with renal syndrome in Jiangxi province, China. Sci Rep. 2020; 10(1): 14291. https://doi.org/10.1038/s41598-020-70761-0 PMID:

32868784

15. Liu XQ, Zhang TC, Xie CY, Xie Y. Changes of HFRS incidence caused by vaccine intervention in Yichun City, China, 2005-2013. Med Sci Monit. 2016; 22: 295-301. https://doi.org/10.12659/msm.895886 PMID: 26818778

16. Ma CF, Yu PB, Nawaz M, Zuo SQ, Jin TZ, Li YL, et al. Hantaviruses in rodents and humans, Xi'an, PR China. J Gen Virol. 2012; 93(Pt 10): 2227-2236. https://doi.org/10.1099/vir.0.043364-0 PMID: 22837422

17. Smith MF, Patton JL. Variation in mitochondrial cytochrome b sequence in natural populations of South American akodontine rodents (Muridae: Sigmodontinae). Mol Biol Evol. 1991; 8(1): 85-103. https://doi.org/10.1093/oxfordjournals.molbev.a040638 PMID: 2002767

18. Liu SW, Xiong Y, Li JX. A duplex quantitative real-time RT-PCR assay for Hantaan virus and Seoul virus. Chin J Zoonoses. 2021; 37: 478-483(in Chinese). https://doi.org/10.3969/j.issn.1002-2694.2021.00.067

19. Wang H, Yoshimatsu K, Ebihara H, Ogino M, Araki K, Kariwa H, et al. Genetic diversity of hantaviruses isolated in china and characterization of novel hantaviruses isolated from Niviventer confucianus and Rattus rattus. Virology. 2000; 278(2): 332-345. https://doi.org/10.1006/viro.2000.0630 PMID: 11118357

20. Kumar S, Stecher G, Li M, Knyaz C, Tamura K. MEGA X: Molecular evolutionary genetics analysis across computing platforms. Mol Biol Evol. 2018; 35(6): 1547-1549. https://doi.org/10.1093/molbev/msy096 PMID: 29722887

21. Liu SW, Gong T, Xu G, Shi Y, Li JX, Zhang YN, et al. Complete genome

sequence analysis of hantaan virsu strain AYW89-15 isolated in Jiangxi, China. Chin J Zoonoses. 2017; 33: 1089-1093(in Chinese). https://doi.org/10.3969/j.issn.1002-2694.2017.12.007

22. Xiao JP, Dai JY, Hu JX, Liu T, Gong DX, Li X, et al. Co-benefits of nonpharmaceutical intervention against COVID-19 on infectious diseases in China: A large population-based observational study. Lancet Reg Health West Pac. 2021; 17: 100282. https://doi.org/10.1016/j.lanwpc.2021.100282 PMID: 34611630

23. Wang L, Wang T, Cui F, Zhai SY, Zhang L, Yang SX, et al. Hemorrhagic Fever with Renal Syndrome, Zibo City, China, 2006-2014. Emerg Infect Dis. 2016; 22(2): 274-276. https://doi.org/10.3201eid/2202.151516 PMID: 26812444

24. Tian H, Tie WF, Li H, Hu X, Xie GC, Du LY, et al. Orthohantaviruses infections in humans and rodents in Baoji, China. PLoS Negl Trop Dis. 2020; 14(10): e0008778. https://doi.org/10.1371/journal.pntd.0008778 PMID: 33075097

25. Zuo SQ, Fang LQ, Zhan L, Zhang PH, Jiang JF, Wang LP, et al. Geo-spatial hotspots of hemorrhagic fever with renal syndrome and genetic characterization of Seoul variants in Beijing, China. PLoS Negl Trop Dis. 2011; 5(1): e945. https://doi.org/10.1371/journal.pntd.0000945 PMID: 21264354

26. Sipari S, Khalil H, Magnusson M, Evander M, Hörnfeldt B, Ecke F. Climate change accelerates winter transmission of a zoonotic pathogen. *Ambio*. 2022; 51(3): 508-517. https://doi.org/10.1007/s13280-021-01594-y PMID: 34228253

27. Zhang X, Chen HY, Zhu LY, Zeng LL, Wang F, Li QG, et al. Comparison of

Hantaan and Seoul viral infections among patients with hemorrhagic fever with renal syndrome (HFRS) in Heilongjiang, China. Scand J Infect Dis. 2011; 43(8): 632-641. https://doi.org/10.3109/00365548.2011.566279 PMID: 21428852

28. Kang YJ, Zhou DJ, Tian JH, Yu B, Guo WP, Wang W, et al. Dynamics of hantavirus infections in humans and animals in Wuhan city, Hubei, China. *Infect Genet Evol*. 2012; 12(8): 1614-1621. https://doi.org/10.1016/j.meegid.2012.07.017 PMID: 22910184

29. Li JL, Ling JX, Liu DY, Liu J, Liu YY, Wei F, et al. Genetic characterization of a new subtype of Hantaan virus isolated from a hemorrhagic fever with renal syndrome (HFRS) epidemic area in Hubei Province, China. *Arch Virol*. 2012; 157(10): 1981-1987. https://doi.org/10.1007/s00705-012-1382-z PMID: 22718221

30. Wang W, Wang MR, Lin XD, Guo WP, Li MH, Mei SH, et al. Ongoing spillover of Hantaan and Gou hantaviruses from rodents is associated with hemorrhagic fever with renal syndrome (HFRS) in China. PLoS Negl Trop Dis. 2013; 7(10): e2484. https://doi.org/10.1371/journal.pntd.0002484 PMID: 24147168

31. Knust B, Brown S, de St Maurice A, Whitmer S, Koske SE, Ervin E, et al. Seoul virus infection and spread in United States home-based ratteries: Rat and human testing results from a multistate outbreak investigation. J Infect Dis. 2020; 222(8): 1311-1319. https://doi.org/10.1093/infdis/jiaa307 PMID: 32484879

32. Cao ZW, Zuo SQ, Gong ZD, Zhan L, Bian CL, Zhang PH, et al. Genetic analysis of a hantavirus strain carried by Niviventer confucianus in Yunnan province,

China. Virus Res. 2010; 153(1): 157-160. https://doi.org/10.1016/j.virusres.2010.06.011 PMID: 20600393

33. Lin XD, Wang W, Guo WP, Zhang XH, Xing JG, Chen SZ, et al. Cross-species transmission in the speciation of the currently known murinae-associated hantaviruses. J Virol. 2012; 86(20): 11171-11182. https://doi.org/10.1128/JVI.00021-12 PMID: 22855492

Supporting information

S1 table. The information of 92 HFRS cases.

S2 table. Reference sequences of orthohantavirus used for analysis in this study.

S3 table. Sequence information of orthohantaviruses obtained in this study.

Click here to access/download
Supporting Information
S1-S3.doc
