# Supplementary material for: Orthohantavirus infections in humans and rodents in the Yichun region, China, from 2016 to 2021
Source: PLoS Negl Trop Dis. 2023 Aug 8;17(8):e0011540. doi: 10.1371/journal.pntd.0011540 (PMC10437993; doi:10.1371/journal.pntd.0011540)
Supplement: Attachment — Submitted filename: response to reviewers.doc [file pntd.0011540.s009.doc]

November 30, 2026

Dear Bruce A. Rosa:

Thank you very much for your prompt treatment of our manuscript, which we submitted for publication as a research article in **PLOS Neglected Tropical Diseases.**

Manuscript No: **PNTD-D-23-00074**

Manuscript title: **Orthohantavirus infections in humans and rodents in the Yichun region, China, from 2016 to 2021**

We would like to sincerely thank the two reviewers through you for their valuable comments and constructive suggestions. We have tried our best to revise our manuscript according to the valuable suggestions. Attached please find the revised version, which we would like to submit for your kind consideration. The changes made in the revised version have been highlighted in YELLOW.

The following is the point-by-point response to the comments and a list of changes we have made. Thank you very much.

With our best wishes,

Yours sincerely,

Shi-Wen Liu

Zhong-Er Long

**Responses to Reviewer 1**

The authors express sincere thanks to you for your valuable comments and constructive suggestions. The following is our point-by-point response to the comments:

**Comment 1: The MS should be revised by a native English speaker.**

Response: The MS has been revised under the direction of a native English speaker.

**Comment 2: line 58, add rodent-associated before viruses.**

Response: “rodent-associated” was added before viruses in line 66.

**Comment 3: lines 74 and 77, The Yichun region? or The Yichun city?**

Response: The Yichun region.

**Comment 4: line 106, add collection after Samples**

Response: We have added the “collection” after “Samples”. (Line 119)

**Comment 5: line 143 and others, the primers sequences should be listed in a table.**

Response: The primer sequences have been listed in S1 Table.

**Comment 6: Fig 2C and 2D, virus infection in rodents and humans detected in this study should be presented in another figure.**

Response: This has been done. The figure of the virus infection in rodents and humans in this study has been modified to Fig 2, Figure 3, and S1 Fig.

**Comment 7: line 253, P should be italicized.**

Response: We have checked and italicized all “P” in the manuscript.

**Comment 8: line 156, add S before segment**

Response: We have added an “S ” before “segment”.(Line 166)

**Comment 9: line 263, were the real-time RT-PCR products sequenced?**

Response: No. The authors thank the reviewer for pointing this out. In our initial manuscript, real-time RT-PCR products were not sequenced. When we were revising the manuscript this time, in response to the reviewer's feedback, we tried to amplify the partial S segment to clarify the genetic characteristics of SEOV in human infection. However, our attempts failed in two SEOV-infected patients (GAHu11/2017, SGHu01/2020). We, therefore, sent the real-time RT-PCR products of these patients to Sangon Biotech Co. for TA cloning and sequencing. As a result of this additional step, we succeeded in obtaining two SEOV sequences (104 nt, GenBank accession no.: OQ731603, OQ731604).

**Comment 10: Line 265, were Dabieshan orthohantavirus (DBSV) identified by primers for HTNV or SEOV? please clarify.**

Response: The complete S segment of DBSV had been amplified using the primers HV-SF and HV-SR, and the partial M segment of DBSV had been amplified using the primers HTNV-F and HTNV-R primers. (Lines 276-278)

**Comment 11: lines 276-277, "The cytochrome b gene sequences of rodents were deposited into GenBank under accession no. OQ187773-OQ187792." should be shown in line 240. In line 240, the rodent species identified by *cytb* gene should be added.**

Response: According to the reviewer’s suggestion, we revised this sentence. Among 33 rodents with orthohantaviruses antigen in lung samples, a total of 20 rodents were confirmed with the cytb gene sequencing methods for species identification, the other 13 rodents were not analyzed for lack of samples. The results were consistent with that of the morphological identification, and their cytb gene sequences were deposited into GenBank under accession no. OQ187773-OQ187792. (Lines 254-258)

**Comment 12: The description for genetic analysis and phylogenetic trees should be revised.**

Response: The description for genetic analysis and phylogenetic trees has been revised and highlighted in yellow.

**Comment 13: rodent name: full genus name at first mention, later with abbreviated genus name throughout the text (exception: at the beginning of a sentence)**

Response: We have checked and revised the abbreviations to ensure that all abbreviations are written out completely when they are first used.

**Comment 14: Partial S should be amplified by PCR. Particularly, the genetic characteristic of SEOV infected in humans should be clarified.**

Response: Partial S segment from samples that failed in complete S segment sequencing had been amplified. We recovered 23 partial S segments from HTNV-infected patients by a nested PCR. We also recovered 2 SEOV sequences from patients which clarified the SEOV infection using the real-time RT-PCR products. The genetic analysis showed that the gene sequences from the two patients infected with SEOV were more related to the widely prevalent SEOV strains than the new variants of SEOV from rodents in this area. This makes us predict that the genetic variants of SEOV from rodents may have weaker pathogenicity. However, more research work is needed in the future to clarify the pathogenicity of the new variants of SEOV circulating rodents in this area.

**Response to Reviewer 2**

The authors express sincere thanks to the reviewer for his/her valuable comments and constructive suggestions. The following is our point-by-point response to the comments:

**Comment 1: Line 42. I suggest inverting the order of summer and winter, because the summer is early:¨in winter and early summer¨**

Response: We have inverted the order of summer and winter in the whole manuscript.

**Comment 2: Line 56-57. Please develop these ideas further: which hosts are known? what species? where do these rodents live? are they exotic or native? wild or commensal?** **expand introduction.**

Response: Orthohantaviruses are carried and transmitted by small animals, including rodents, shrews, and bats. There were 38 species of orthohantavrius according to the Current Taxonomy of ICTV. Each hantavirus species usually appears to be primarily associated with one host species. We list one example of each kind of host (rodent, shrew, and bat) to answer these questions and expand the introduction. For instance, HTNV has been demonstrated in *A. agrarius* in Far East Russia, China, and South Korea. SEOV harbored by *R. norvegicus* is present worldwide. Huangpi virus has been found in bats (*Pipistrellus abramus*), and Cao Bang virus in shrews (Anourosorex squamipes) in China. Transmission among nature hosts and from hosts to human generally occurs via aerosolized infected excreta. Rodents are the predominant natural hosts and the source of human infection. In rodents, these viruses typically cause asymptomatic persistent infections that cause no apparent harm. However, in humans, rodent-associated viruses cause HFRS in Eurasia and cardiopulmonary syndrome in North and South America. (Lines 57-68)

**Comment 3: Line 70-73. Please, join these two sentences in only one.**

Response: We have joined these two sentences in one: Several orthohantavirus species can cause HFRS, including HTNV, SEOV, Puumala orthohantavirus, and Dobrava-Belgrade orthohantavirus, and the predominate causative pathogens of HFRS in China were HTNV and SEOV. (Lines 78-81)

**Comment 4: Line 74-75. Please, move to methods.**

Response: We have moved this sentence to the methods part. (Lines 105-106)

**Comment 5: Line 77-79. Please, join these two sentences.**

Response: We have joined these two sentences in one: Despite this situation, no molecular surveys of orthohantaviruses have been carried out in the Yichun region, and the circulating pathogens of HFRS in humans and rodents remained unknown. (Lines 86-89)

**Comment 6: Line 81. Avoid this type of words in the ephigraphes.**

Response: Fig 1 was revised and the title was rewritten as “The study area (the Yichun region) and the sampling sites”.

**Comment 7: Line 82-88. This paragraph is more methods than introduction. Please reorder the ideas and write a clear objective in this part and move the methods details to the following section.**

# Response: The objective of this study was to investigate the epidemiology features of HFRS across the Yichun region from 2016 to 2021 and to clarify the causative pathogens in both humans and rodents. The findings of the study will provide crucial information for developing effective HFRS control strategies, not only in the Yichun region but also in other areas across China（Lines 90-94）. The methods details were moved to the method section.

**Comment 8: Line 99. locations of the human cases?**

Response: The human cases were composed of all the reported HFRS cases across the Yichun region from 2016 to 2021 (Fig 1).

**Comment 9: Line 108. This section need more information: number of taps, trapping-effort, bait, type of trap, number of nights? Please, complete. In these season in all of studied years? Are the same sites every year or change?**

Response: Rodents were trapped in cages at 5- meter intervals and baited with peanuts. The trap sites included field and residential areas (Line 123-124). We trapped rodents in three counties in the Yichun region, Gaoan, Tonggu, and Shanggao (Fig1). Gao`an is an old and serious HFRS area in the Yichun region, the rodent samples were collected in spring and fall/winter in all of the studied years and at the same sites every year. Samples in Tonggu and Shanggao were in these seasons and at the same sites but not in all the studied years. The trapping effort in Gaoan County was 200 rodents per year (2016-2021). The trapping effort in Tonggu was 50 rodents per year (2017-2021), and the trapping effort in Shanggao was 50 rodents per year (2018-2020). The total taps from 2016 to 2021 were 9600, 8000, 8600, 6810, 6740, and 8270 respectively.

**Comment 10: Line 112. How did the rodents authenticated?**

Response: The rodents were initially classified into specific species by morphological examination during trapping, and those that showed the presence of orthohantavirus antigen were confirmed by cytb gene analysis in the laboratory. （Lines 124-128）Among 33 rodents with orthohantaviruses antigen in lung samples, a total of 20 rodents were confirmed with the *cytb* gene sequencing methods for species identification, the other 13 rodents were not analyzed for lack of samples. The results were consistent with that of the morphological identification, and their cytb gene sequences were deposited into GenBank under accession no. OQ187773-OQ187792. (Lines 254-259)

**Comment 11: Line 114-116. Please, indicate these areas in a map. In the same map that you have to indicate the endemic areas and rodent trapping areas.**

Response: Sampling sites were indicated in Fig 1.

**Comment 12: Line 117. I suggest move to result section.**

Response: Thanks for the reviewer’s suggestion. It has been moved to the result section. (Line 237)

**Comment 13: Line 130. move to the previous section.**

Response: It has been moved to the previous section. (Lines 126-128)

**Comment 14: Line 206-207. specify and highlight the data that must be observed, it is not enough to insert a table with results if the text does not indicate what should be seen from it.**

Response: Thanks for pointing this out. We have added and revised the description for the table description. (Lines 216-219)

**Comment 15: Line 211. considerer the option: ¨....and one case per month ocurred in March, May, June and September.¨**

Response: We thank the reviewer for this suggestion. The sentence was rewritten as: Of the 11 deaths, three occurred in January, two occurred in November, two occurred in December, and one case per month occurred in March, May, June, and September. (Lines 222-223)

**Comment 16: Line 215. The epigraph have to explain what type of information are in the table or graph. Please, rewrite.**

Response: We rewrite the epigraph as “The case number and morbidity rate of HFRS in the Yichun region (2016–2021)”. (Line 224)

**Comment 17: Line 217. Please, rewrite like this: Number of HFRS cases, (B) Monthly distribution of HFRS cases reported on CISDCP, (C) virus infection rate in rodents (black) and HFRS mortality in humans (red) and (D) Monthly distribution of orthohantavirus from 92 patients with HFRS in the Yichun region (2016–2021). Please, copy this style to all epigraphs.**

Response: We rewrote the title of the figure according to the suggestion. (Lines 226-227)

**Comment 18: Line 222. Specify and highlight the data that must be observed, it is not enough to insert a table with results if the text does not indicate what should be seen from it.**

Response: We have checked and revised the text for the table description. (Lines 230-232)

**Comment 19: Line 224. Move to methods and explain why you decided these categories. 16-60 years is a big interval that mix a lot of people with diverse activities.**

Response: We reanalyzed the age distribution of HFRS cases via very five years intervals and informed the result in **S2 Table**. Then, we divided all the cases into five age groups in the revised manuscript, including <16, 16-30, 31-35, 46-60, and >60 years of age group (Table 2).

**Comment 20: Line 225. I suggest inform the number of cases/year of age, because is obvious that the more width interval have the higher number of cases.**

Response: The authors thank the reviewer for this suggestion. We reanalyzed the age distribution of HFRS cases via very five years intervals and informed the result in **S2 Table.**

**Comment 21: Line 240. This is methods.**

Response: We moved the sentence “Viral infection in rodents was analyzed by orthohantavirus antigen detection.” to the method section. (Line 135)

**Comment 22: Line 249. Add a line with the trapping effort per year. Without this data I cannot compare the values among years.**

Response: The total taps from 2016 to 2021 were 9600, 8000, 8600, 6810, 6740, and 8270, respectively (Lines 261-262).

**Comment 23: Line 250, 253. Add the p-value and add the analyses methods in the corresponding section.**

Response: The p-value (0.473) was added in line 264. The analysis methods were added in the method section: Data of HFRS patients and rodents were analyzed with Excel 2007. Statistical analysis was performed using IBM SPSS Statistics software, version 21. The inter-group difference in the virus infection rates was compared by using the Chi-square test. A value of P<0.05 was considered significant (Lines 204-207).

**Comment 24: Line 258. Please, copy the epigraph style of the previous table.**

Response: We revised the title of Table 4 as the reviewer`s suggestion: “Orthohantvirus in rodents by species in the Yichun region (2016–2021)”

**Comment 25: Line 271-273. It is the objective and Methods, not results**

Response: We revised and moved this sentence to the method section. (Lines 167-169)

**Comment 26: Line 335. Please, explain how these two diseases are related to prevent hantavirus transmission. How are HFRS transmitted? The transmission to human is not inside the houses? please, expand this topic.**

Response: Humans can become infected with orthohantaviruses through the inhalation of aerosolized excreta from infected rodents. The most effective way to control orthohantaviruses disease is to reduce human exposure to infected rodents and excrement. With the improvement in housing conditions, rodents are no longer commonly found inside people's houses in this area. As a result, outdoor exposure is the most probable way to be infected. The lower epidemic of HFRS in 2020 and 2021 might have been due to the control measures and advice for COVID-19, such as staying home, opening windows, wearing a mask when going out, and cleaning hands frequently. These measures could have reduced the inhalation not only of SARS-CoV-2 but also of orthohantaviruses. (Lines 354-362)

**Comment 27: Line 341. Why? the HPS peaks are in summer, for example. Which is the difference between both hantaviroses?**

Response: Studies showed that the peak of HFRS associated with SEOV occurred in spring, whereas the peak of HFRS associated with HTNV occurred mainly in winter. However, HTNV is the sole source of HFRS in Xi’an and Baoji, China, winter and summer peaks of HFRS cases have been reported. In the present study, the winter and early summer peaks were all caused by HTNV. We believe that the characteristics of peaks are not due to the differences in virus species, but rather to the intensity of exposure and climate. In this study, farmers were the predominant population and the Yichun region is a large agricultural region in Jiangxi Province with a subtropical monsoon climate. *A. agrarius*, the major carrier of HTNV, was the predominant rodent in the wild. From spring to late autumn, local farmers spend most of their time planting cereals and vegetables in the field, favoring higher chances of contacting *A. agrarius* and inhaling their body fluids containing HTNV. With an incubation period ranging from 7 to 60 days, the peaks of HFRS can occur in early summer and winter. As for the climate reasons. According to the research by Saana Sipari et al, climate change can alter the transmission intensity within the host population, thus may influence human infection and changing the peaks. Of course, the climate-induced mechanisms are poorly understood (Lines 367-382).

**Comment 28: Line 342. add possible reasons.**

Response: Farmers constituted the predominant population suffering from HFRS. Because the Yichun region is a large agricultural region in Jiangxi Province. From spring to late autumn, local farmers spend most of their time planting cereals and vegetables in the field, favoring higher chances of contacting rodents and inhaling their body fluids containing hantaviruses.(Lines 373-379)

**Comment 29: Line 343. I am repeating, this age class is very width.**

Response: We reanalyzed the age distribution of HFRS cases. we divided all the cases into five age groups in the revised manuscript: <16, 16-30, 31-35, 46-60, and >60 years of age. The top age groups for the number of cases are 46-60 years old, then followed by the over 60 years group, this may be due to the tremendous number of young people leaving rural areas to work in cities, while the middle-aged and elderly adults tended to stay in villages and engaged in more agricultural activities making them more susceptible to HFRS infection.(Lines388-392)

**Comment 30: Line 345. expanded this idea.**

Response: protective measures including hats, boots gloves, goggles, face masks, and coveralls in agricultural operations may reduce exposure to infected rodents and excrement. (Lines 393-394)

**Comment 31: Line 353. detail how is the transsmition.**

Response: Humans can become infected with orthohantaviruses through the inhalation of aerosolized excreta from infected rodents (Lines 354-355).

**Comment 32: Line 354. why?**

Response: As for the climate reason, according to the research by Saana Sipari, et al, climate change can alter the transmission intensity within the host population, thus may influence human infection and changing the peaks. However, the climate-induced mechanisms are poorly understood. （Lines 380-383）

**Comment 33: Line 355-356. Describe the biology of these species that could help understand the importance in the transmission to human.**

Response: *A. agrarius* in the wild and *R. norvegicus* in the residential area were the major carriers of HTNV and SEOV, respectively. The HTNV sequences recovered from HFRS cases were closely related to those from *A. agrarius*, suggesting that the new genetic variant of HTNV is directly transmitted from rodents to humans. （Lines 421-422）

**Comment 34: Line 358. reservoirs or spill-overs? Discuss**

Response: Those are natural reservoirs of orthohantavirus, and no host spill-over event was found in this study. （Lines 399-400）

**Comment 35: Line 373. May be is due different biology of the hosts. Discuss this.**

Response: The authors thank the reviewer for pointing this out. This reminds us that the genetic variants of SEOV circulated in rodents in this area may have weaker pathogenicity because no genetic variants of SEOV strains were detected in the patients. The gene sequences from the only two patients infected with SEOV were more closely related to the widely prevalent SEOV strains than the new variants of SEOV circulating in rodents in this area (Lines 404-407). We should strengthen the surveillance of SEOV in patients to determine its epidemic status in humans.
