# Supplementary material for: Orthohantavirus infections in humans and rodents in the Yichun region, China, from 2016 to 2021
Source: PLoS Negl Trop Dis. 2023 Aug 8;17(8):e0011540. doi: 10.1371/journal.pntd.0011540 (PMC10437993; doi:10.1371/journal.pntd.0011540)
Supplement: Attachment — Submitted filename: response to reviewers.docx [file pntd.0011540.s010.docx]

July 14, 2023

Dear Bruce A. Rosa:

Thank you very much for your prompt treatment of our manuscript, which we submitted for publication as a research article in **PLOS Neglected Tropical Diseases.**

Manuscript No: **PNTD-D-23-00074R1**

Manuscript title: **Orthohantavirus infections in humans and rodents in the Yichun region, China, from 2016 to 2021**

We would like to sincerely thank two reviewers through you for the positive feedback and further suggestions. We have tried our best to revise our manuscript according to the valuable suggestions. Attached please find the revised version, which we would like to submit for your kind consideration. The changes made in the revised version have been highlighted in YELLOW.

The result of the formatting check by PLOS Neglected Tropical Diseases Staff reminded us there might be copyright issues with one figure (original Fig 1 in R1), which is a map of the study area and the sampling sites. To avoid copyright issues, we removed the figure in the revised manuscript and marked these areas with longitude and latitude in the text. These changes did not affect the results, conclusions of this article, or the readers' understanding of this article.

The following is the point-by-point response to the comments and a list of changes we have made. We hope our manuscript is suitable for publication after the above revisions.

Thank you very much.

With our best wishes,

Yours sincerely,

Shi-Wen Liu

Zhong-Er Long

**The following is the response to the comments from Reviewer #3:**

Response: The authors express sincere thanks to the reviewer for his/her valuable comments and constructive suggestions.

1. It remains unclear what was the reason for using the neighbor-joining method for phylogenetic analysis.

Response: We tried to construct the phylogenetic trees by Neighbor-joining and Maximum likelihood methods, and the resulting phylogenetic trees were similar. Because all the sequences we analyzed belong to orthohantavirus with a small evolutionary distance, and the length of the gene sequence we analyzed is very short, especially for some partial S segments, we ultimately used the neighbor-joining method for analysis. The neighbor-joining method is a commonly used method for physiological analysis. The relevant information has been added to lines 421-426 of the revised manuscript.

2. Analysis of amino acid sequences would be more informative compared to nucleotide sequences. That is because some nucleotide substitutions may not change amino acids resulting in limited changes in protein function. Also, without the comparison of amino acid sequences, the statement of finding new lineages of Hantaan orthohantavirus has limited support.

Response: We have added the comparative analysis of amino acids in the revised manuscript (highlighted in yellow, lines 288-316). According to the analysis of deduced amino acids sequence of the complete S CDS area (429 aa), the similarity among the Nineteen strains of HTNV and other known HTNV reference strains is 95.8%-99.3%, and there are 11 to 16 amino acid variations compared to HTNV standard reference strain 76-118. Analysis of the partial M segment (amino acid positions from 655 to 753) of HTNV showed that 58 strains in this study shared 89.9%-98.9% amino acid identities with other known HTNV strains and a unique amino acid variation (Ser700Gly). Based on comparative analysis of nucleotide and amino acid sequences as well as phylogenetic analysis, we believe that the Hantaan orthohantavirus (HTNV) belong to new lineages.

3. Strain SGHu02/2020 has different locations on S and M segment phylogenetic trees. How authors could explain this?

Response: The different locations of Strain SGHu02/2020 on S and M segment phylogenetic trees may be due to the relatively conservative S segment, and the sequence of the S segment we analyzed is shorter (222 nt) than the M segment (298 nt), with fewer nucleotide variations. Another possible reason is that the orthohantavirus RNA genome consists of three independent segments and the segment recombination events could happen in nature. The S or M segment of SGHu02/2020 may have undergone a recombination event, however, the gene sequences that we obtained are limited and we are unable to conclude recombination. In the future, we will pay more attention to the recombination events. The relevant information has been added to lines 426-435 of the revised manuscript.

4. Public relevance should be clearly stated.

Response: Hemorrhagic fever with renal syndrome (HFRS) caused by orthohantavirus is a serious public health problem in Eurasia. In this study, we investigated the epidemiology features of HFRS across the Yichun region, a high-incidence area of HFRS, and cleared the causative pathogens in both humans and rodents. We identified new genetic variants of SEOV, HTNV, and DBSV from rodent lung samples in the Yichun region, which shows the high genetic diversity of the orthohantaviruses. Special attention should be paid to the high genetic diversity of orthohantavirus when developing vaccines and antiviral drugs. We also found the new genetic variant of HTNV in rodents caused human infection and was the main prevalent virus of human infection in the Yichun region. These results will provide very important information for HFRS control in the Yichun region and other areas in the world (Lines 44-51).
